# The Issue of Misidentification of Kojic Acid with Flufuran in *Aspergillus flavus*

**DOI:** 10.3390/molecules24091709

**Published:** 2019-05-02

**Authors:** Marina DellaGreca, Gaetano De Tommaso, Maria Michela Salvatore, Rosario Nicoletti, Andrea Becchimanzi, Mauro Iuliano, Anna Andolfi

**Affiliations:** 1 Department of Chemical Sciences, University of Naples ‘Federico II’, 80126 Naples, Italy; dellagre@unina.it (M.D.); gaetano.detommaso@unina.it (G.D.T.); mariamichela.salvatore@unina.it (M.M.S.); mauro.iuliano@unina.it (M.I.); 2 Council for Agricultural Research and Economics, Research Centre for Olive, Citrus and Tree Fruit, 81100 Caserta, Italy; rosario.nicoletti@crea.gov.it; 3 Department of Agriculture, University of Naples ‘Federico II’, 80055 Portici, Italy; andrea.becchimanzi@unina.it

**Keywords:** kojic acid, flufuran, 5-(hydroxymethyl)-furan-3-carboxylic acid, *Aspergillus flavus*, bees, immunomodulation

## Abstract

In the course of investigations on the complex phenomenon of bee decline, *Aspergillus flavus* was isolated from the haemocoel of worker bees. Observations on the metabolomic profile of this strain showed kojic acid to be the dominant product in cultures on Czapek-Dox broth. However, an accurate review of papers documenting secondary metabolite production in *A. flavus* also showed that an isomer of kojic acid, identified as 5-(hydroxymethyl)-furan-3-carboxylic acid and named flufuran is reported from this species. The spectroscopic data of kojic acid were almost identical to those reported in the literature for flufuran. This motivated a comparative study of commercial kojic acid and 5-(hydroxymethyl)-furan-3-carboxylic acid, highlighting some differences, for example in the ^13^C-NMR and UV spectra for the two compounds, indicating that misidentification of the kojic acid as 5-(hydroxymethyl)-furan-3-carboxylic acid has occurred in the past.

## 1. Introduction

Fungi have evolved the capability to produce a great number of secondary metabolites involved in the improvement of their ecological fitness, and many of them play important biological roles as virulence factors, chemical defense agents, developmental regulators, insect attractants, and chemical signals for communication with other organisms. On these properties is founded the pharmacological exploitation of many products as antibiotic, antiviral, antitumor, antihypercholesterolemic, and immunosuppressant agents [1,2,3,4,5]. In this respect, fungi are prime targets of a vigorous investigational activity, based on the employment of the most advanced analytical and structure elucidation techniques [6,7].

A wide array of fungal secondary metabolites has been ascribed to the ascomycete genus *Aspergillus*, well-known for its ubiquity and cosmopolitan distribution [7,8]. The species *Aspergillus flavus* is well-known as a foodstuff contaminant and a mycotoxin producer, and in this respect its metabolomic profile has been quite well characterized [9,10]. However, this fungus has been also reported in association with plants and animals in many different environments [11,12]. Particularly, it has been directly isolated from bee (*Apis mellifera*) individuals in different developmental stages and health conditions [13]. Although reported as the agent of stonebrood disease, further elucidation is required if *A. flavus* basically behaves like an entomopathogen, or if its relationships with bees are more complex and eventually involve modulation of the immunitary response to other pathogens and parasites of these insects. Generally, the fungus is considered an opportunistic pathogen of immunocompromised individuals, gaining access to the host through ingestion, or taking advantage from the interaction of bees with other pathogens and parasites which negatively affect host immunocompetence and cuticle integrity [13,14,15,16].

During investigations on the honeybee colony collapse, a multifactorial syndrome mainly related to the compresence of immunosuppressive viruses and *Varroa destructor*, a parasitic mite which is also known as a possible vector [16,17,18], strains of *A. flavus* were repeatedly isolated from the haemocoel of worker bees.

The present paper reports the identification of kojic acid (KA) as the main metabolite obtained from this source. Furthermore, revision of the structure of flufuran, previously characterized as 5-(hydroxymethyl)furan-3-carboxylic acid from *A. flavus* and other fungal species is proposed, based on a comparison of spectroscopic data of commercially available compounds and some derivatives.

## 2. Results

### 2.1. Comparison of NMR Data Obtained from KA *(**1**)* and 5-(Hydroxymethyl)furan-3-carboxylic Acid *(**2**)*

A white solid was obtained from the extraction with ethyl acetate (EtOAc) of the culture filtrate of strain AB1EET of *A. flavus*, which consisted in a main metabolite, as deduced by its ^1^H and ^13^C-NMR spectra. NMR data collected for the extracted compound showed significant similarities with proton and carbon chemical shifts reported in literature for both KA (**1**) [19] and 5-(hydroxymethyl)furan-3-carboxylic acid (**2**) [20], a compound characterized from *A. flavus* and other fungal species (Table 1). A full analysis of the previous reports revealed discrepancy between the carbon chemical shifts of furan compounds [21] and those assigned for 5-(hydroxymethyl)furan-3-carboxylic acid. In order to clarify this issue, commercially available KA and 5-(hydroxymethyl)furan-3-carboxylic acid were submitted to spectroscopic and potentiometric investigations.

**Figure 1:**
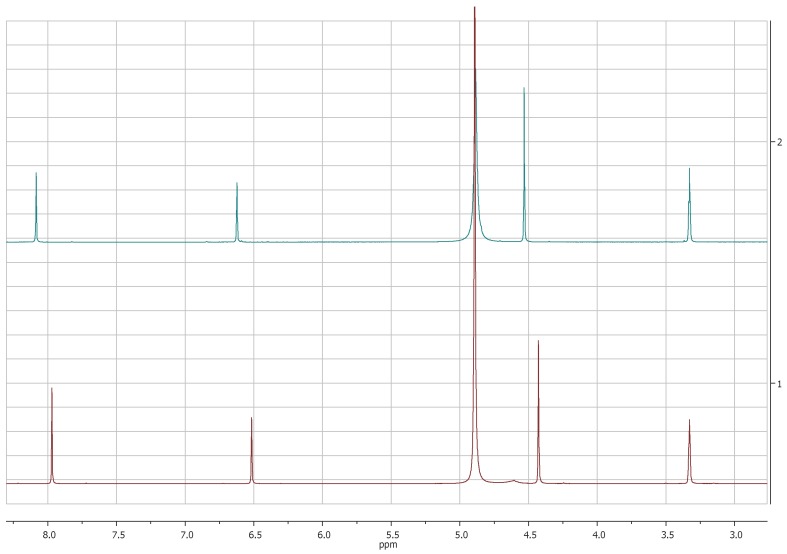
^1^H-NMR spectra of KA (**1**, red) and 5-(hydroxymetyhyl)-furan 3-carboxylic acid (**2**, green) recorded at 400 MHz in CD_3_OD.

**Figure 2:**
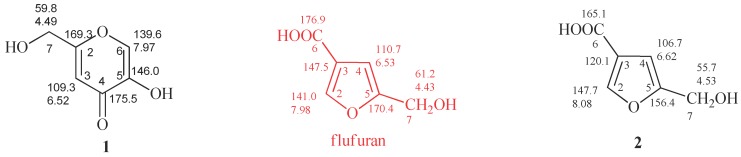
Proton and carbon chemical shifts of KA (**1**) and 5-(hydroxymethyl)furan-3-carboxylic acid (**2**). Data reported in red were erroneously assigned to flufuran. The spectra were recorded in CD_3_OD.

**Figure 3:**
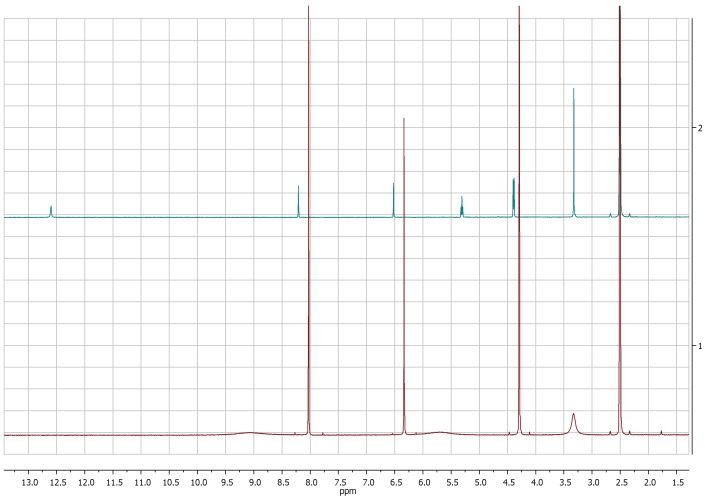
^1^H-NMR spectra of KA (**1**, red) and 5-(hydroxymetyhyl)-furan 3-carboxylic acid (**2**, green) recorded at 400 MHz in DMSO*d_6_*.

The comparison of ^1^H-NMR spectra, recorded in CD_3_OD, showed no significant differences between 5-(hydroxymethyl)furan-3-carboxylic acid and KA (Figure 1 and Figure 2). Notwithstanding, the ^1^H-NMR spectra recorded in DMSO*d*_6_ for **2** revealed the coupling between the proton of the hydroxyl group in C-7 and protons of the methylene group which resonate as triplet and doublet, respectively, at δ 5.31 and 4.40 (*J* = 5.8 Hz) and the presence of the signal of proton of the carboxylic group at δ 12.60 (Figure 3).

The main differences between **1** and **2** were observed, as expected, in ^13^C-NMR spectra. The C-3, C-5 and C-6 carbons of **2** resonate at δ 120.1, 156.4 e 165.1 (Figure 2 and Figure S1), respectively, assigned by 2D-NMR spectra (Figures S2 and S3). However, the ^13^C-NMR spectrum of flufuran showed chemical shift values identical to KA (Figure 2), indicating a misinterpretation of the flufuran structure.

### 2.2. Comparison of Potentiometric, UV, and MS Spectrophotometric Measurements

**Figure 4:**
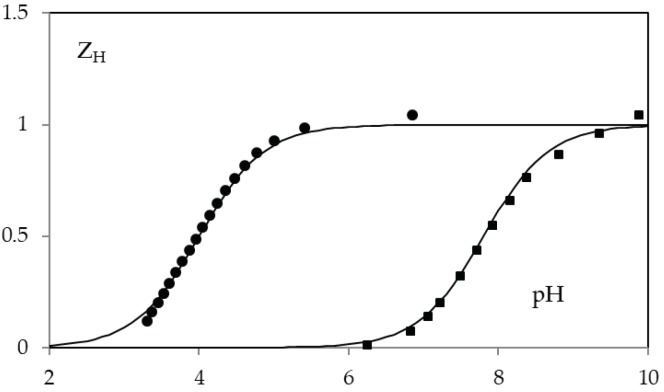
*Z*_H_(pH) function in 0.1 M NaClO_4_ for KA (squares) and 5-(hydroxymethyl)furan-3-carboxylic acid (circles). The solid lines are calculated with protonation constants cologarithm value 7.68 (on squared) and 4.03 (on circles).

The acid-base behavior of KA and 5-(hydroxymethyl)furan-3-carboxylic acid was determined by potentiometric and spectrophotometric methods. The calculated protonation constants cologarithm values are 7.68 ± 0.05 and 4.03 ± 0.05, for compound **1** and **2** respectively. Potentiometric data are reported in Figure 4. 

In particular KA constant is very similar to that in 0.1 M KCl (7.7) [33]. While no value on the protonation of 5-(hydroxymethyl)furan-3-carboxylic acid had been previously reported.

UV spectra of the pure compounds, recorded in water in the interval 200–400 nm, show λ_max_ at 216 and 270 nm for KA, and at 240 nm for **2**. Similar values were observed when the spectra were recorded in methanol.

**Figure 5:**
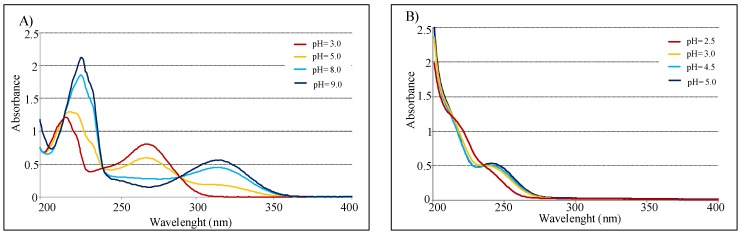
(**A**) UV spectra of 2.0 × 10^−3^ M in 0.1 M NaClO_4_ for KA. (**B**) UV spectra of 2.0 × 10^−3^ M in 0.1 M NaClO_4_ for 5-(hydroxymethyl)furan-3-carboxylic acid (optical path 0.2 cm).

In agreement with acid-base behavior of both compounds, measurements conducted in pH function confirmed that KA spectra are highly influenced by pH [34]. In fact, absorbance value at 315 nm increases from 3 to 9 pH range, instead the peak at 270 nm decreases in the same interval. Conversely, UV spectra of compound **2** present minimal variation in function of pH (Figure 5). Finally with pH increasing, KA shows a isosbestic point at 285 nm, while 5-(hydroxymethyl)furan-3-carboxylic acid presents one at 240 nm.

The fragmentation pattern observed in MALDI TOF/MS spectra evidenced relevant analogies between the two compounds, as to be expected from considering the nature of the respective functional groups. In fact, from peaks corresponding to the protonated ions [M+H]^+^ at *m*/*z* 143 of **1** and **2**, fragments deriving from the loss of OH, CHO, and COOH groups were observed in both cases.

### 2.3. Comparison of KA and 5-(Hydroxymethyl)furan-3-carboxylic Acid Derivatives

Considering reports concerning the presumptive isolation of the 7-*O*-acetyl derivative of flufuran [25] and of its methyl ester [29], in this work the differences between derivatives of **1** and **2** obtained from common acetylation and methylation reaction were also evaluated.

**Figure 6:**
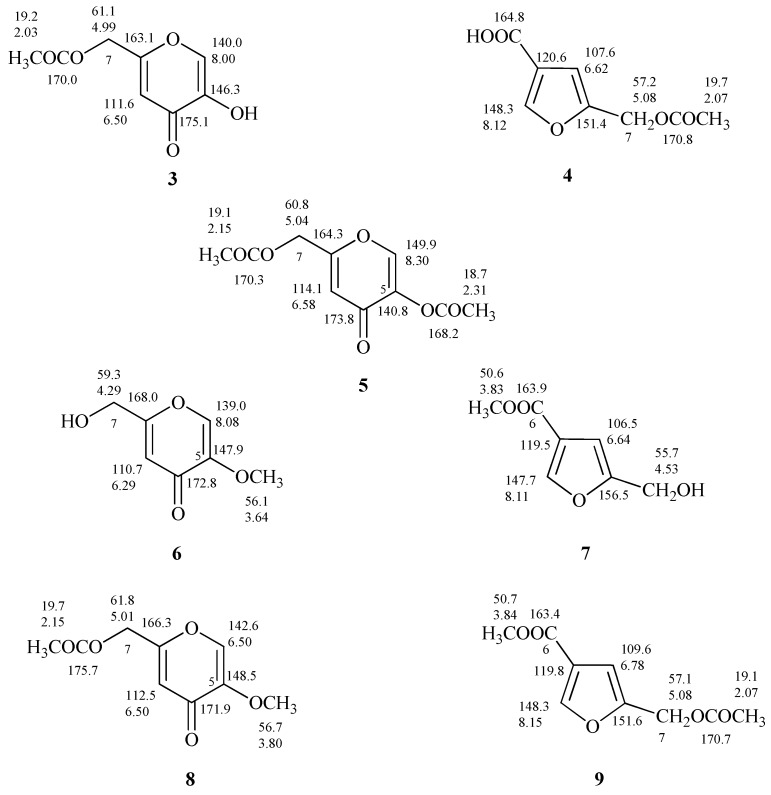
Proton and carbon chemical shifts of 7-*O*-acetylKA, 5,7-*O,O’*-diacetylKA, 5-*O*-methylKA, 5-*O*-methyl-7-*O’*-acetylKA (**3**, **5**, **6**, and **8**); 5-(acetoxymethyl)furan-3-carboxylic acid, methyl 5-(hydroxymethyl)furan-3-carboxylate, methyl 5-(acetoxymethyl)furan-3-carboxylate (**4**, **7**, and **9**).

The proton spectrum of 5-(acetoxymethyl)furan 3-carboxylic acid (**4**, Figure S4), produced after acetylation of **2**, showed the down shift of Δδ 0.55 of the signal assigned to the methylene group C-7 which resonates as singlet at δ 5.08, and the presence of a further singlet at δ 2.07 assigned to the CH_3_ of the acetyl group (Figure 6 and Figure S4). As discussed in relation to **1** and **2** the differences between the ^1^H-NMR spectra of **4** and 7-*O*-acetylflufuran [25] are not so marked compared to the ones of ^13^C-NMR spectra (Figure 6 and Figure S5).

From the data reported on the diacetylated flufuran [22], it is possible to exclude its formation from **2**; most likely the described compound is 5,7-*O,O′*-diacetylKA (**5**, Figure 6), derived from **1**.

The treatment of **1** and **2** with diazomethane led to the production of 5-*O*-methylKA (**6**) and methyl 5-(hydroxymethyl)furan-3-carboxylate (**7**, Figure 6) as deduced from ^1^H and ^13^C-NMR spectra. In fact, NMR spectra of **7** (Figures S6 and S7) showed further singlets, respectively, at δ 3.83 and at δ 50.6 attributed to methoxy group. As observed for the other compounds under examination, the comparison of ^13^C-NMR spectra of methyl derivatives of **1** and **2** revealed significant differences (**6** and **7**, Figure 6).

Finally, the preparation of 5-*O*-methyl-7-*O’*-acetylKA (**8**) and methyl 5-(acetoxymethyl)furan-3-carboxylate (**9**) and the interpretation of their one-dimensional NMR spectra (Figures S8 and S9) allowed to confirm the derivative structures, and to establish that the derivative i.e., methyl 5-(acetoxymethyl)furan-3-carboxylate) reported by Evidente et al. [22] is undoubtedly **8**.

## 3. Discussion

The furan compound 5-(hydroxymethyl)furan-3-carboxylic acid was identified for the first time as a natural product in cultures of *Polyporus ciliatus* [20], and afterwards reported from other fungi (Table 1). Although furan derivatives are not an extensive class of fungal compounds [35], over the past few years the literature concerning these natural products has been enriched by new reports [36,37,38,39,40]. According to their biosynthetic origin, the structure of natural furan derivatives shows the presence of furan rings substituted in position 2 and 5 [35]. Flufuran represented an exception to such a general model.

In the present paper, we produce evidence that in most of the previous reports the product identified as flufuran was instead KA. In addition to being known as a typical secondary metabolite of both *A. flavus* and *A. oryzae*, KA has been reported from several *Penicillium* spp. [41]. Other fungal species are claimed as producers of some derivatives [42], indicating that biosynthetic ability for this compound might be more widespread. To the best of our knowledge, our amendments to previous findings represents the first evidence of its production in important fungal genera, such as *Fusarium* and *Pestalotiopsis*, which deserve further confirmation also with reference to correct taxonomic identification of producing strains. In view of the present revision, the bioactivities erroneously assigned to flufuran should be instead referred to KA, integrating the known profile of biological properties of this γ-pyrone compound [43]. Actually, these data show that KA and 5-(hydroxymethyl)furan-3-carboxylic acid are susceptible of misidentification. Particularly, some instrumental techniques commonly used for the identification of secondary metabolites in fungal extracts (i.e., ^1^H-NMR and ESI MS data) are not able to distinguish between these two compounds. However, huge differences can be observed between **1** and **2** in the UV spectra obtained with different solvents and pH. The acid-base properties are also valid to differentiate between **1** and **2**. Finally, observations concerning acetyl and methyl derivatives also confirmed the misidentification of KA with flufuran.

## 4. Materials and Methods

### 4.1. General Experimental Procedures

^1^H and ^13^C-NMR spectra were recorded at 400 and at 100 MHz, respectively, in CD_3_OD unless otherwise noted, on a Bruker spectrometer (AscendTM400) (Bremen, Germany); the same solvent was used as internal standard. Potentiometric titrations were performed in an air-bath thermostat kept at (25.00 ± 0.05) °C. A programmable computer-controlled Data Acquisition Unit 34970A, (Agilent Tecnologies Inc., Santa Clara, CA, USA) was used to perform the potentiometric measurements. The glass electrodes were Metrohm (Herisau, Switzerland) of 60102-100 type, and Ag/AgCl electrode was utilized as reference. The EMF values were measured with a precision of ± 0.01 mV using a Keithley 642 type Digital Electrometer (Tektronix Inc., Beaveron, OR, USA). UV–VIS spectra were recorded by model Cary 5000 Spectrophotometer by Varian (Palo Alto, CA, USA) from 200 to 600 nm (optical path 0.2 cm) at 25.0 °C, under a constant flow of nitrogen. 

MALDI-TOF/MS spectra were acquired by a 4700 Proteomics Analyzer (Applied Biosystems, Framingham, MA, USA). Compounds were detected in reflector mode using α-cyano-4-hydroxycinnamic acid (CHCA) as matrix.

Analytical and preparative TLC were performed on silica gel plates (Kieselgel 60, F_254_, 0.25 mm) (Merck, Darmstadt, Germany). The spots were visualized by exposure to UV radiation (253 nm), or by spraying with 10% H_2_SO_4_ in MeOH followed by heating at 110 °C for 10 min. 5-(hydroxymethyl)furan-3-carboxylic acid and KA were purchased from Enamine Ltd. (Kyiv, Ukraine) and Alfa Aesar (Karlsruhe, Germany), respectively.

### 4.2. Isolation of *A. flavus* from Bees

Worker bee individuals were collected from experimental apiaries at the Department of Agriculture, University of Naples Federico II (Portici, Italy) in October–November 2018. Freshly emerged bees (<48 h) were anaesthetized by chilling on ice for 20 min, and surface-disinfected by dipping in 70% ethanol for 30 s. The intersegmental membrane between head and thorax was cut using a sterile scalpel. Ten microliters of the liquid matter exuding from haemocoel were aseptically collected using a micropipette, and spotted on potato-dextrose agar (PDA, Oxoid) amended with 50 mg/L chloramphenicol. Plates were incubated at 32 °C in the dark. Hyphal tips from the emerging fungal colonies were transferred to fresh PDA plates to obtain pure cultures for morphological identification and storage. The strains obtained were readily identified as belonging to the *Aspergillus* section *Flavi*, based on the formation of colonies producing a dense felt of yellow-green rough conidiophores with radiate heads, and subglobose echinulate conidia. An orange reverse could be observed in cultures on a specific medium (AFPA) [44]. Unequivocal ascription to the species *A. flavus* resulted after rDNA-ITS and calmodulin gene sequencing. To this purpose, total genomic DNA was extracted from fresh mycelium taken from pure culture of strain AB1EET using CTAB protocol [45]. According to reported methodology, primers ITS1F and ITS4 were used to amplify rDNA-ITS, while primers CF1M and CF4 were used to amplify the calmodulin gene [45,46]. The original DNA sequences obtained in this study have been deposited in GenBank under the codes MK611561 (ITS) and MK611938 (calmodulin). The calmodulin gene sequence proved to be more respondent for unequivocal species identification, yielding 100% homology with sequences from 60 strains of *A. flavus* available in GenBank.

### 4.3. Production and Extraction of KA

Strain AB1EET was cultured in Czapek-Dox broth (Oxoid) following a previously reported procedure [47]. The freeze-dried culture filtrates (750 mL) were dissolved in 100 mL, acidified with HCl 2 N at pH 3, and extracted three times with an equal volume of EtOAc. Organic phases were combined, dried with Na_2_SO_4_, and evaporated under reduced pressure originating a white solid residue (350.7 mg) identified as KA.

### 4.4. Determination of Protonation Constants

The evaluation of the protolysis constants was conducted through spectrophotometric and potentiometric titration, at 25 °C in 0.1 M NaClO_4_ as ionic medium [48]. By experimental data the average number (*Z*_H_) of protons released per molecule was assessed through the equation:

*Z*_H_ = ([H^+^] − *C*_H_ − K_w_/[H^+^])/*C*_L_ (1)

where [H^+^] is hydrogenionic concentration, *C*_H_ is the total acid concentration, *C*_L_ is the compound concentration and K_w_ is ionic product (10^−13.7^, in 0.1 M NaClO_4_). Experimental data were processed with Hyperquad softare [49]. 

### 4.5. UV and MS Data of KA and 5-(Hydroxymethyl)furan-3-carboxylic Acid

Kojic acid (**1**). UV λ_max_ nm (log ε): (H_2_O) 216 (4.12), 270 (3.93); (MeOH) 225 (3.99), 270 (3.99); (pH 3.0) 215 (3.47), 270 (3.30); (pH 5.0) 216 (3.51), 268 (3.17), 318 (2.63); (pH 8.0) 227 (3.66), 315 (3.5); (pH 9.0) 227 (3.72), 315 (3.15). MALDI TOF/MS: *m*/*z* 143 [M+H]^+^, 125 [M-OH]^+^, 113 [M-CHO]^+^, 97 [M-COOH]^+^, 69 [M-CH_2_OH-COO]^+^. 

5-(Hydroxymethyl)furan-3-carboxylic acid (**2**). UV λ_max_ nm (log ε): (H_2_O) 240 (3.39); (MeOH) 240 (3.38); (pH 2.5) 243 (2.97); (pH 3.0) 243 (3.07); (pH 4.0) 243 (3.07); (pH 5.0) 243 (3.12). MALDI TOF/MS: *m/z* 143 [M+H]^+^, 125 [M-OH]^+^, 113 [M-CHO]^+^, 97 [M-COOH]^+^.

### 4.6. Sample Methylation

Fifteen milligrams of samples [KA, 5-(hydroxymethyl)furan-3-carboxylic acid, 5-(acetoxymethyl)furan-3-carboxylic acid (**1**, **2** and **4**)] were dissolved in MeOH (1.5 mL); an ethereal solution of CH_2_N_2_ was slowly added until a yellow color became persistent. The reaction mixtures were stirred at room temperature for 4 h. The solvent was evaporated under a N_2_ stream at room temperature. Residues of each reaction were analyzed by TLC on silica gel; **6**, was evidenced at Rf 0.37 by eluting with EtOAc-MeOH (9:1), while Rf 0.54 and 0.82 corresponded to **7** and **9** respectively, as eluted with CHCl_3_-i-PrOH (92:8).

### 4.7. Sample Acetylation

Ten mg of samples [KA, 5-(hydroxymethyl)furan-3-carboxylic acid, 5-*O*-methylkojic acid (**1**, **2**, **6**)], dissolved in pyridine (30 μL), were converted into the corresponding acetyl derivatives (**5, 4, 7**) by acetylation with Ac_2_O (30 μL) at room temperature overnight. The reaction was stopped by addition of MeOH, and the azeotrope formed by addition of benzene was evaporated in a N_2_ stream at 40 °C. Residues of each reaction were analyzed by TLC on silica gel; **5** was evidenced at Rf 0.44 by eluting with CHCl_3_-i-PrOH (95:5), while Rf 0.54 and 0.82 corresponded to **7** and **9** respectively, as eluted with CHCl_3_-i-PrOH (92:8).

## Figures and Tables

**Table 1:** Previous reports dealing with flufuran identification.

Source	Spectroscopic Data	Activity	Ref.
*Aspergillus flavus*	^1^H and ^13^C-NMR, MS, IR, UV	Antifungal	[22]
*A. flavus*	—	Cytotoxic against human cancer cell lines	[23]
*A. flavus*		Bacteriostatic (*Escherichia coli*)	[24] ^1^
*A. flavus* ^2^	^1^H and ^13^C-NMR	Antibacterial, antioxidant	[25]
*A. flavus*	MS	—	[26]
*Aspergillus oryzae*	—	—	[27]
*A. oryzae*	^1^	Antioxidant, antimicrobial	[28]
*Fusarium sp.* ^3^	^1^H and ^13^C-NMR	Antimicrobial	[29]
*Penicillium dipodomyicola*	^1^H and ^13^C-NMR	—	[30]
*Penicillium polonicum*	—	Monoamine oxidase inhibitor	[31]
*Pestalotiopsis sp.*	^1^H and ^13^C-NMR	Cytotoxic against HepG2 cells	[32]
*Polyporus ciliatus*	P.f., MS, ^1^H and ^13^C-NMR, MS, IR, UV	—	[20]

^1^ This paper is in Chinese and could not be examined in detail. ^2^ 7-*O*-Acetyl derivative was also isolated. ^3^ Methyl ester was also isolated.

## Supplementary Materials

The following are available online, Figures S1–S3. NMR spectra of KA (**1**) and 5-(hydroxymethyl)furan-3-carboxylic acid (**2**); Figures S4–S9. ^1^H and ^13^C-NMR spectra of acetyl and methyl derivatives of **2**.
