# Supplementary material for: The Issue of Misidentification of Kojic Acid with Flufuran in Aspergillus flavus
Source: Molecules. 2019 May 2;24(9):1709. doi: 10.3390/molecules24091709 (PMC6539386; doi:10.3390/molecules24091709)
Supplement: Supplementary file 1 [file molecules-24-01709-s001.pdf]

Supporting Information

The Issue of Misidentification of Kojic Acid with Flufuran in *Aspergillus flavus*

Marina DellaGreca [1], Gaetano De Tommaso [1], Maria Michela Salvatore [1], Rosario Nicoletti [2,3], Andrea Becchimanzi [3], Mauro Iuliano [1] and Anna Andolfi [1,*]

[1] Department of Chemical Sciences, University of Naples 'Federico I' 80126 Naples, Italy; mariamichela.salvatore@unina.it_(M.M.S.); dellagre@unina.it (M.D.); gaetano.detommaso@unina.it (G.deT.); mauro.iuliano@unina.it (M.I:)

[2] Council for Agricultural Research and Agricultural Economy Analysis, 00184 Rome, Italy; rosario.nicoletti@crea.gov.it (R.N)

[3] Department of Agriculture, University of Naples 'Federico II', 80055 Portici, Italy; andrea.becchimanzi@unina.it

* Correspondence: andolfi@unina.it (A.A); Tel.: +39-081-2539179

Figure legend

Figure S1. ^{13}C NMR spectra of KA (**1**, red) and 5-(hydroxymethyl)-furan 3-carboxylic acid (**2**, green) recorded at 100 MHz in CD_3OD.

Figure S2. HSQC spectrum of 5-(hydroxymethyl)-furan 3-carboxylic acid (**2**), recorded in CD_3OD.

Figure S3. HMBC spectrum of 5-(hydroxymethyl)-furan 3-carboxylic acid (**2**), recorded in CD_3OD.

Figure S4. ^{1}H NMR spectra of 5,7-*O,O'*-diacetylKA (**5**, red) and 5-(acetoxymethyl)-furan 3-carboxylic acid (**4**, green) recorded at 400 MHz in CD_3OD.

Figure S5. ^{13}C NMR spectrum of 5-(acetoxymethyl)-furan 3-carboxylic acid (**4**) recorded at 100 MHz in CD_3OD.

Figure S6. ^{1}H NMR spectrum of methyl 5-(hydroxymethyl)furan-3-carboxylate (**7**) recorded at 400 MHz in CD_3OD.

Figure S7. ^{13}C NMR spectrum of methyl 5-(hydroxymethyl)-furan 3-carboxylate (**7**) recorded at 100 MHz in CD_3OD.

Figure S8. ^{1}H NMR spectrum of methyl 5-(acetoxymethyl)furan-3-carboxylate (**9**) recorded at 400 MHz in CD_3OD.

Figure S9. ^{13}C NMR spectrum of methyl 5-(acetoxymethyl)furan-3-carboxylate (**9**) recorded at 100 MHz in CD_3OD.

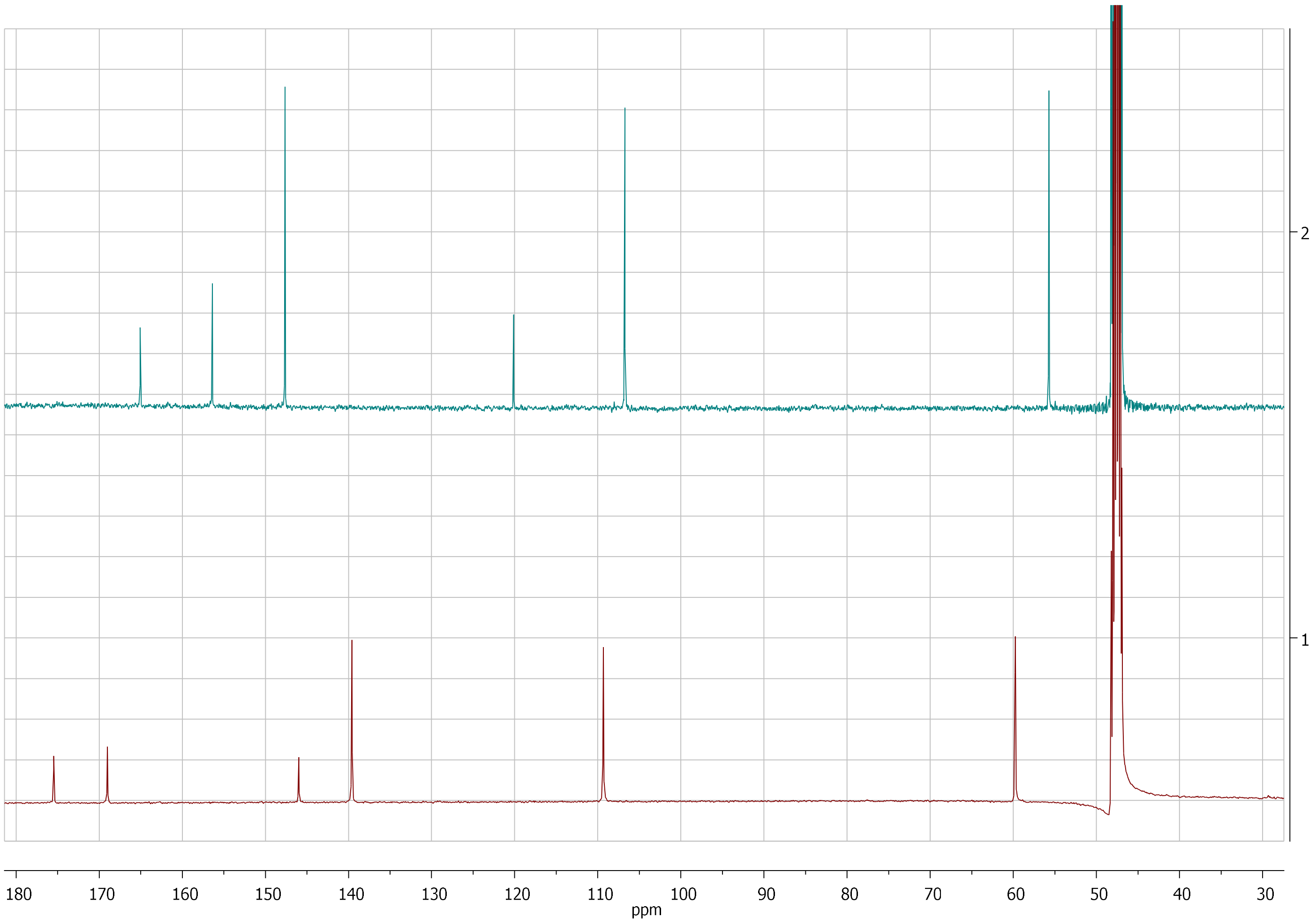

Figure S1. ^{13}C NMR spectra of KA (**1**, red) and 5-(hydroxymethyl)-furan 3-carboxylic acid (**2**, green) recorded at 100 MHz in CD_3OD.

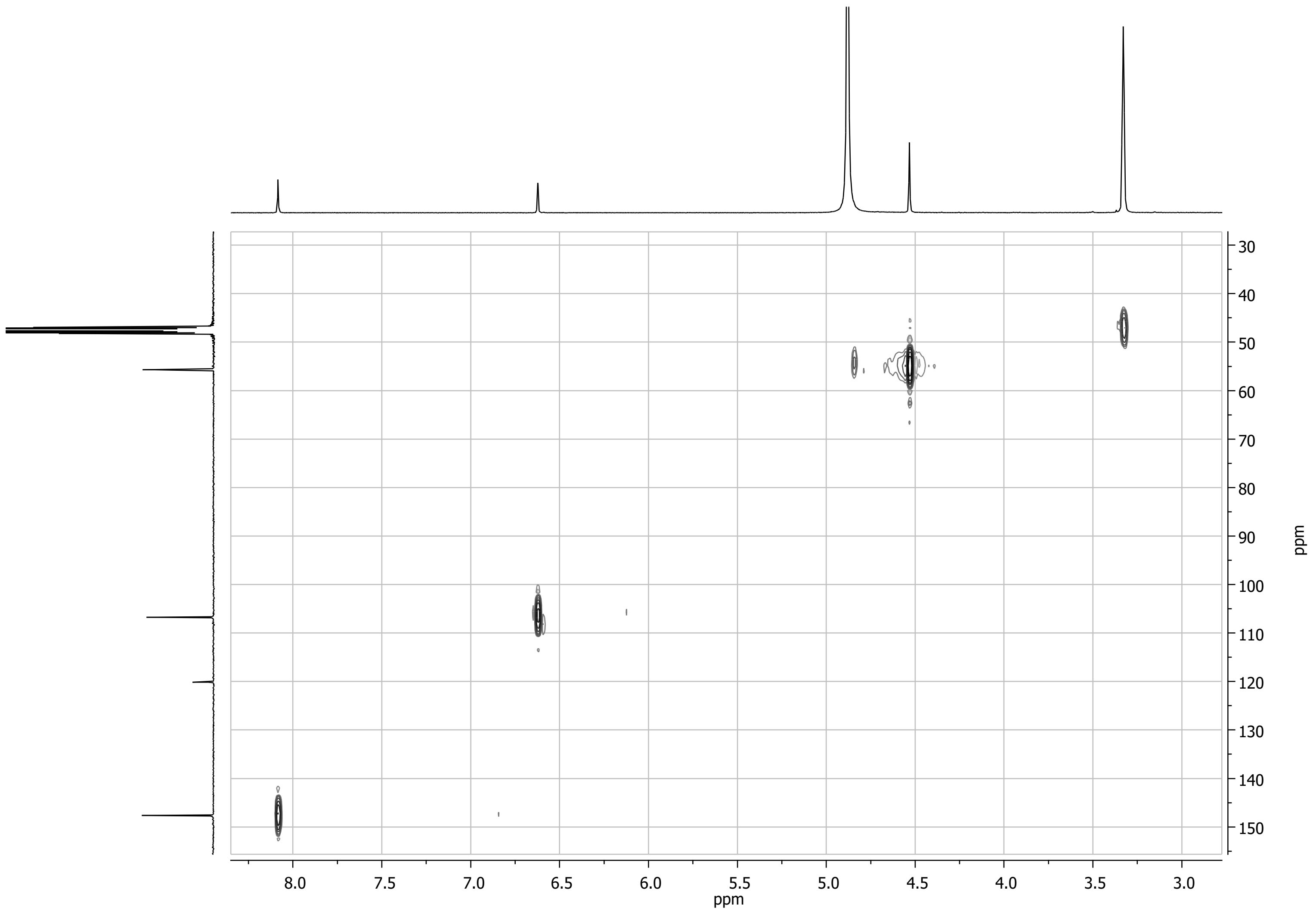

Figure S2. HSQC spectrum of 5-(hydroxymethyl)-furan 3-carboxylic acid (**2**), recorded in CD_3OD.

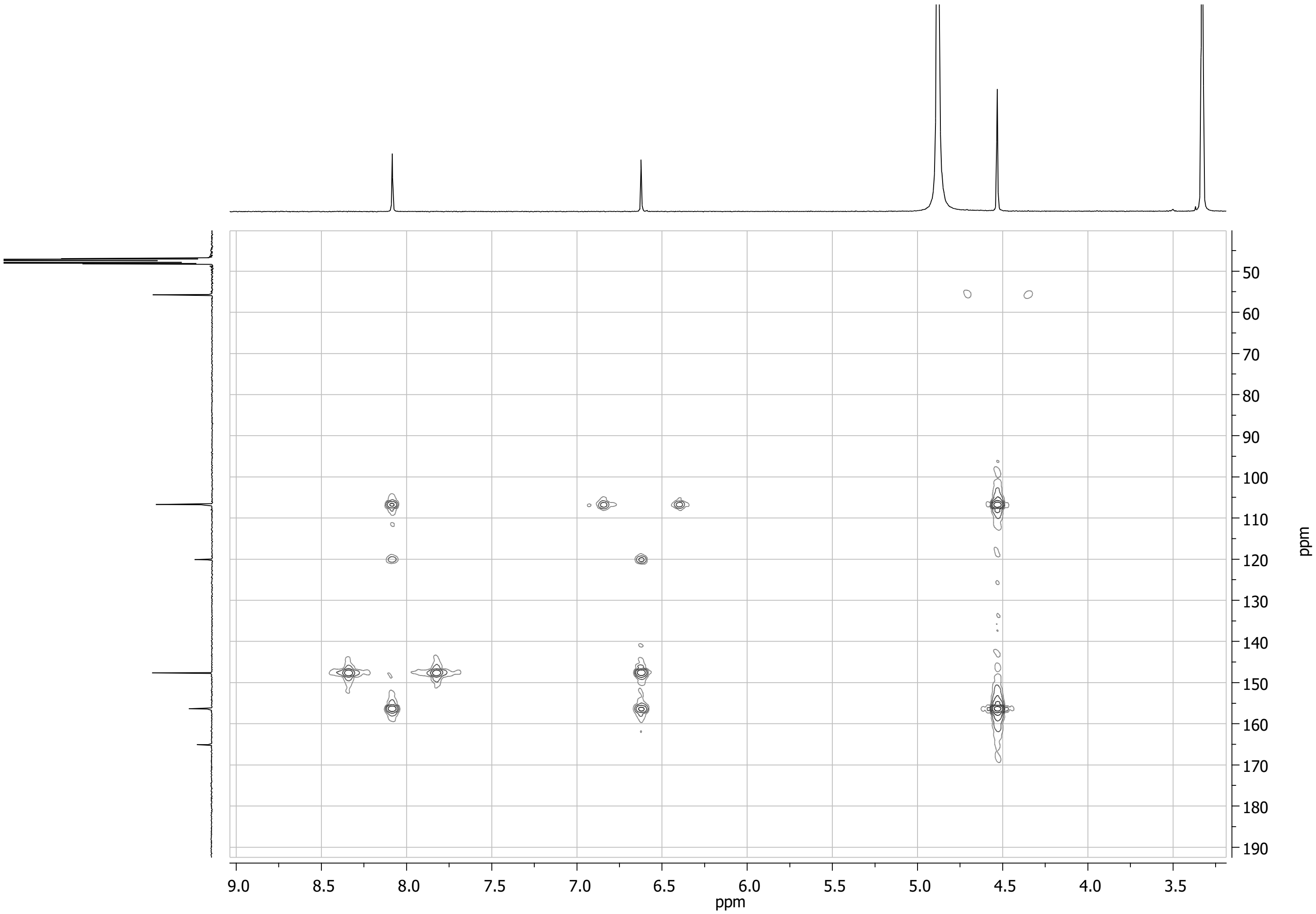

Figure S3. HMBC spectrum of 5-(hydroxymethyl)-furan 3-carboxylic acid (**2**), recorded in CD_3OD.

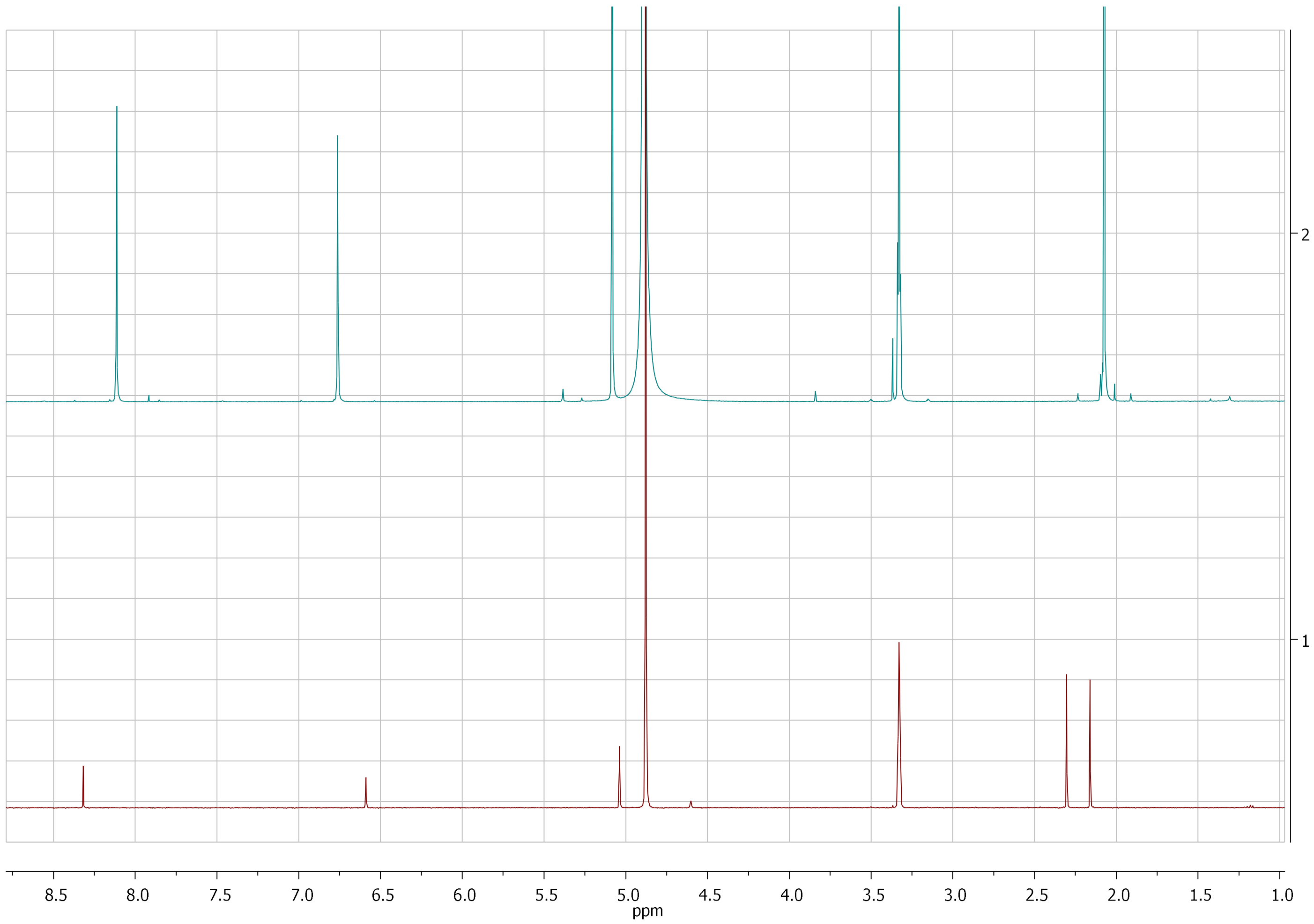

Figure S4. ^{1}H NMR spectra of 5,7-*O*,*O*′-diacetylKA (**5**, red) and 5-(acetoxymethyl)-furan 3-carboxylic acid (**4**, green) recorded at 400 MHz in CD_3OD.

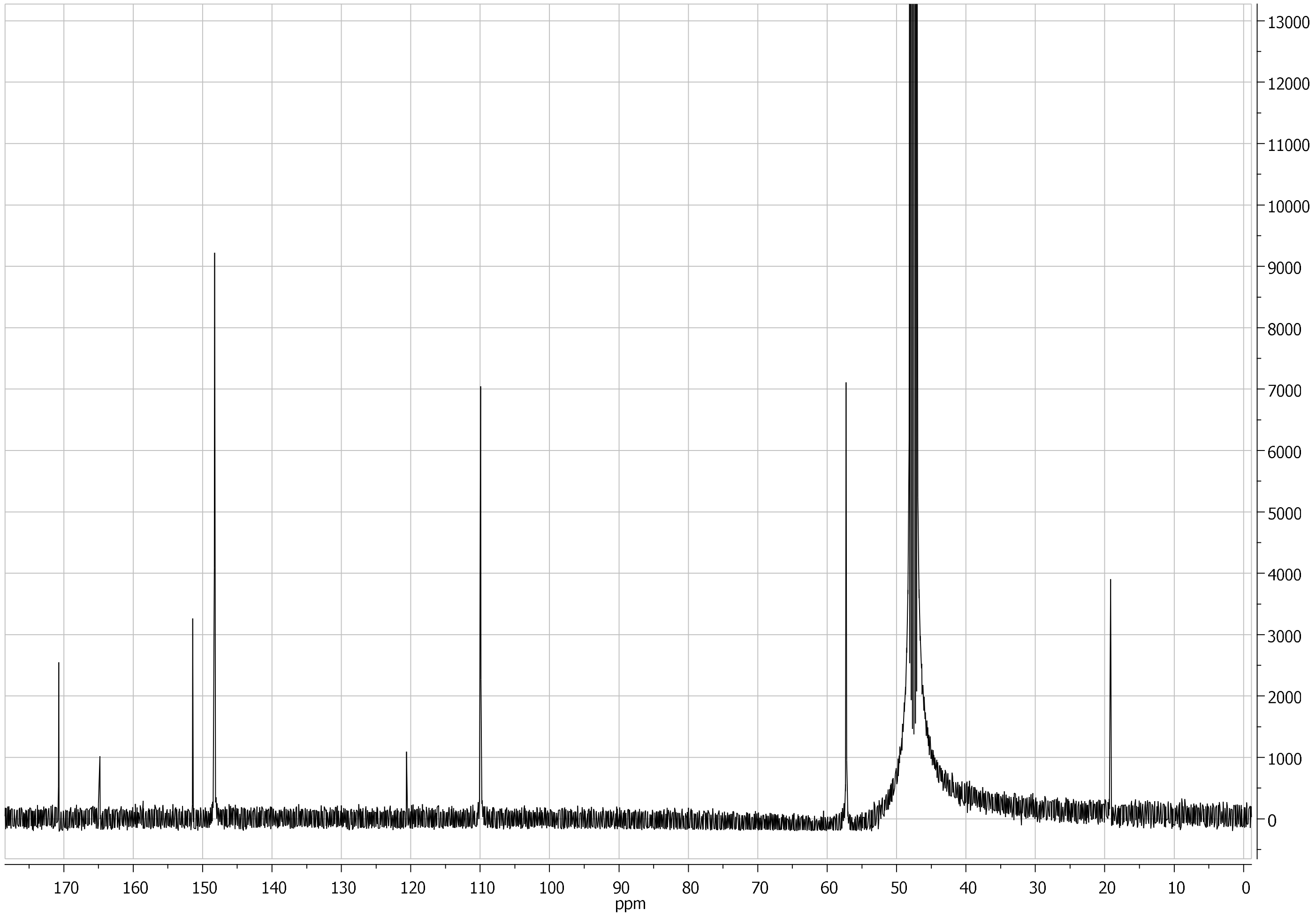

Figure S5. ^{13}C NMR spectrum of 5-(acetoxymethyl)-furan 3-carboxylic acid (**4**) recorded at 100 MHz in CD_3OD.

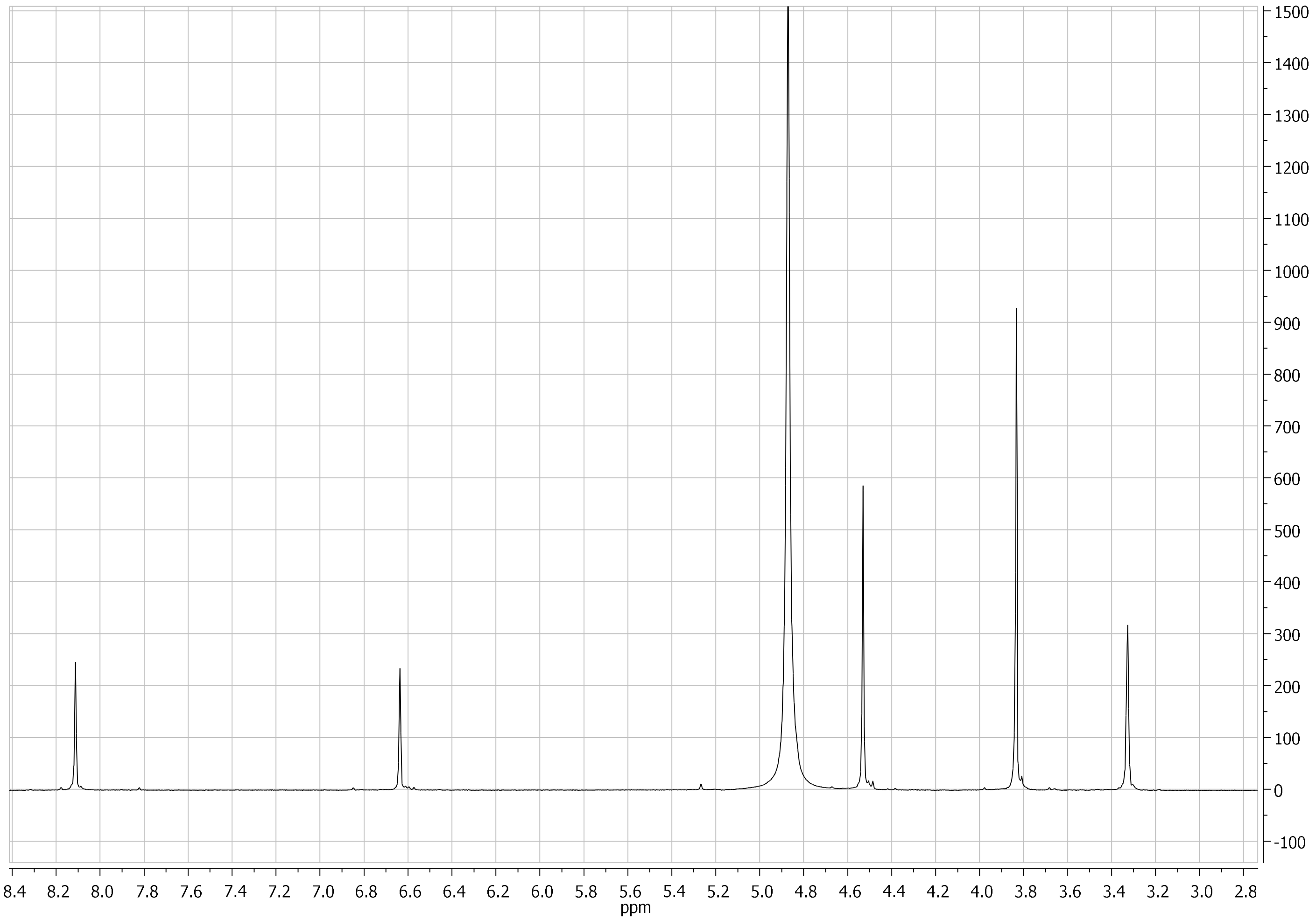

Figure S6. ^{1}H NMR spectrum of methyl 5-(hydroxymethyl)furan-3-carboxylate (**7**) recorded at 400 MHz in CD_3OD.

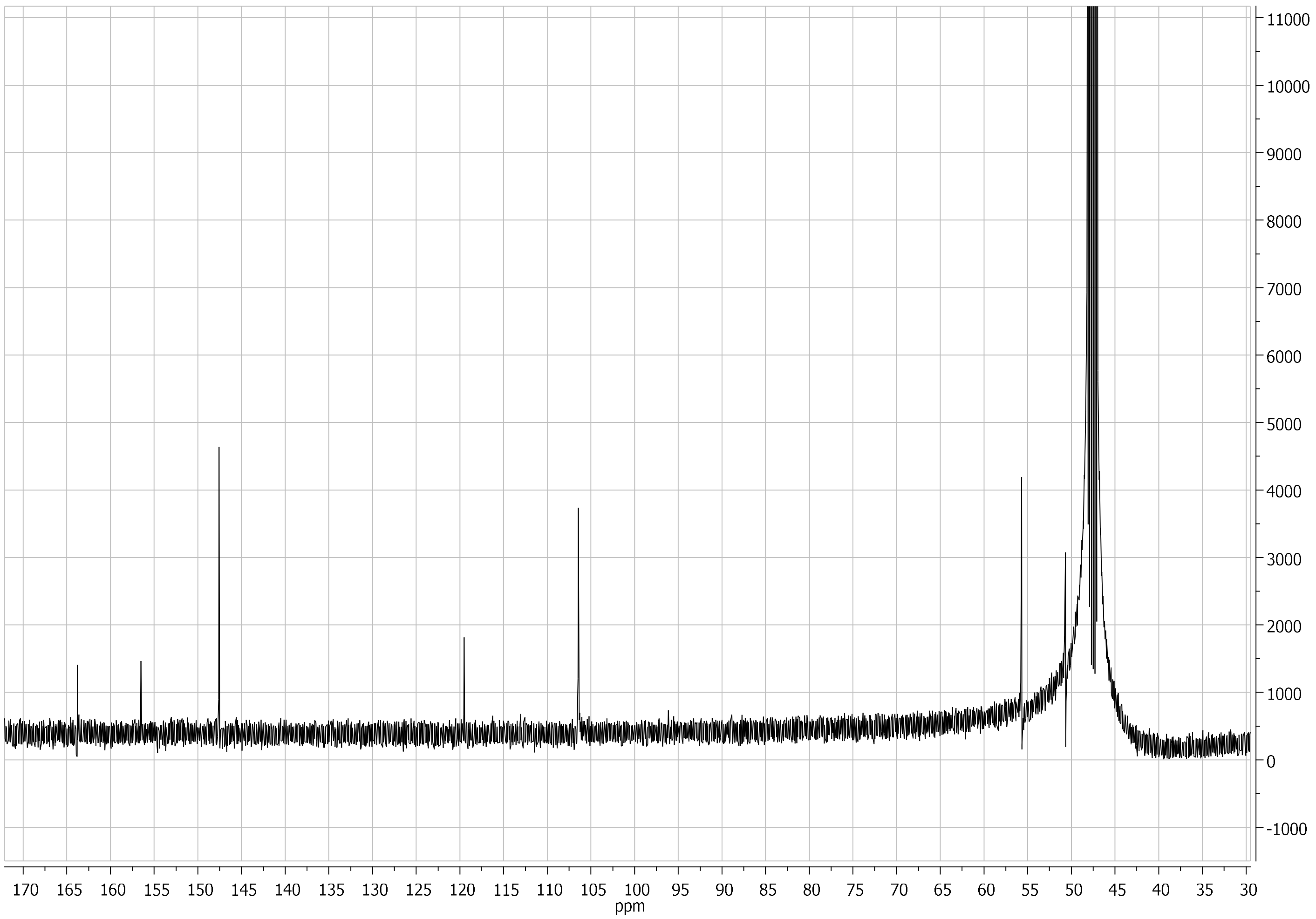

Figure S7. ^{13}C NMR spectrum of methyl 5-(hydroxymethyl)-furan 3-carboxylate (**7**) recorded at 100 MHz in CD_3OD.

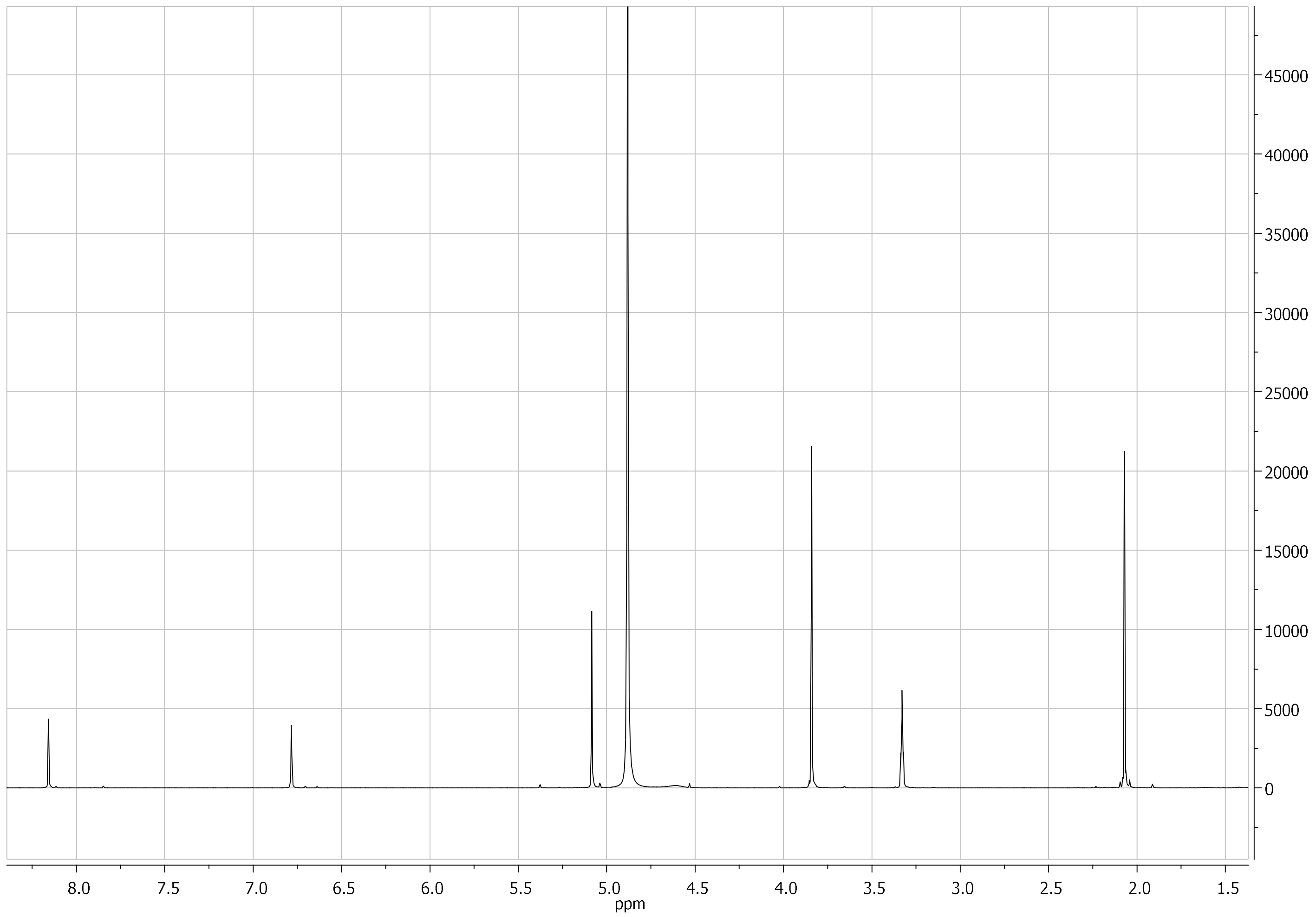

Figure S8. ^{1}H NMR spectrum of methyl 5-(acetoxymethyl)furan-3-carboxylate (**9**) recorded at 400 MHz in CD_3OD.

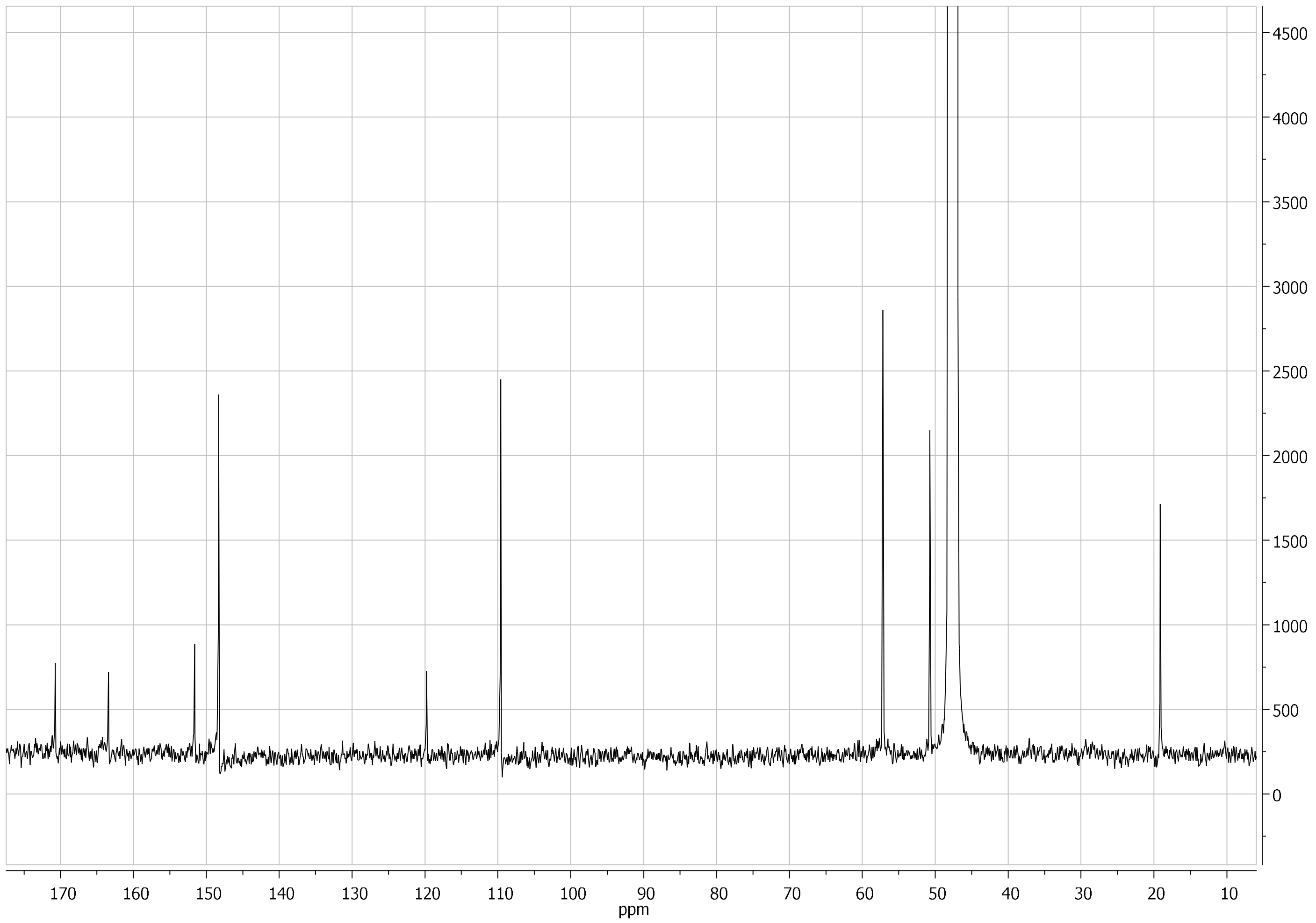

Figure S9. ^{13}C NMR spectrum of methyl 5-(acetoxymethyl)furan-3-carboxylate (**9**) recorded at 100 MHz in CD_3OD.
